# The incidence and risk factors of chronic kidney disease after radical nephrectomy in patients with renal cell carcinoma

**DOI:** 10.1186/s12885-022-10245-8

**Published:** 2022-11-05

**Authors:** Shuai Wang, Zhenghong Liu, Dahong Zhang, Fei Xiang, Wei Zheng

**Affiliations:** 1 Urology & Nephrology Center, Department of Urology, Zhejiang Provincial People’s Hospital, Affiliated People’s Hospital, Hangzhou Medical College, Hangzhou, 310014 Zhejiang China; 2 The Second Clinical Medical College, Zhejiang Chinese Medical University, Zhejiang, Hangzhou China

**Keywords:** Prognosis, Renal cell carcinoma, Radical nephrectomy, Renal dysfunction

## Abstract

**Background:**

Radical nephrectomy (RN) is the standard treatment for localized renal cell carcinoma. The decrease in nephrons from RN could lead to postoperative chronic kidney disease (CKD). In this study, we aim to investigate the incidence and risk factors for CKD in patients who have received RN.

**Methods:**

A Total of 1233 patients underwent radical nephrectomy in Zhejiang Provincial People’s Hospital from January 2010 to December 2018. Those who had an abnormal renal function before surgery or were lost to follow-up were excluded. Five hundred patients were enrolled in the end. eGFR was calculated using the abbreviated MDRD equation. CKD was defined as eGFR less than 60 ml/min/1.73m^2^. The incidence of postoperative CKD was estimated using the Kaplan-Meier method. The independent risk factors for CKD occurrence were determined through logistic multivariate regression analysis.

**Results:**

Patients were followed up for a median of 40 month (3–96 months), with CKD occurring in 189 cases. The 5-year cumulative incidence of CKD was 43.4%. There was a significant difference between these189 patients and the remaining patients without post nephrectomy CKD in terms of age, sex, weight, and preoperative eGFR(*P*<0.05). Multivariate regression analysis showed that age (OR = 1.038, 95%CI = 1.002–1.076), preoperative eGFR of the contralateral kidney (OR = 0.934, 95%CI = 0.884–0.988) and Immediate postoperative eGFR (OR = 0.892, 95%CI = 0.854–0.931) were independent risk factors for postoperative CKD.

**Conclusions:**

The incidence of CKD after radical nephrectomy was not uncommon. Age, preoperative eGFR of the contralateral kidney and Immediate postoperative eGFR are independent risk factors for postoperative CKD.

## Background

Radical nephrectomy (RN) is the historical and standard treatment for localised renal cell carcinoma (RCC) [1]. However, the likelihood of a decline in kidney function after kidney cancer surgery is an important consideration when treating patients with a renal mass. It has been proven that the decrease in nephrons from RN could lead to postoperative chronic kidney disease (CKD) [2]. According to the Kidney Disease Outcome Quality Initiative (K/DOQI) guidelines [3], CKD was defined as a glomerular filtration rate (GFR) or estimated GFR (eGFR) less than 60 mL/min per 1.73 m^2^. There is increasing concern that surgically induced CKD will be associated with an increased risk of hospitalisation, cardiovascular events, and death, as has been shown in CKD from all causes. To the best of our knowledge, long-term follow-up data on CKD incidence in patients post RN are rare. In this study, we sought to determine postoperative long-term changes in renal function after RN in patients with RCC.

## Methods

This research was a retrospective study. The clinical data of 1233 renal cell carcinoma patients who received radical nephrectomy were collected from Jan. 2010 to Dec. 2018. All patients were from Zhejiang Provincial People’s Hospital. Institutional Review Board approval was obtained from the Ethics Committee of Zhejiang Provincial People’s Hospital (ID Num: QT2022331). Patients who had preoperative metastatic disease or preoperative CKD (GFR<60 ml/min/1.73m^2^) and those whose follow-up was less than 3 months were excluded.

Demographic and clinical information such as age, sex, weight, height, profession, BMI, hemoglobin, urine protein, blood lipids, and pathology grade were collected for analysis. Additionally, past histories of smoking, hypertension, diabetes, renal disease, and other clinical characteristics possibly related to CKD were also collected. Serum creatinine (Scr) was used to calculate eGFR, with preoperative GFR measured using dynamic renal scintigraphy. If patient didn’t have dynamic renal scintigraphy before surgery, eGFR was used as an alternative to GFR to assess renal function. Surgery was performed using standardized techniques of open or laparoscopic radical nephrectomy. All patients received routine review after operation, including physical examination, laboratory examination and B-ultrasound or CT examination. Serum creatinine results within one week after surgery, and at one month, three months, six months and annual postoperative follow-up were collected. Immediate postoperative eGFR value was defined as eGFR calculated within one week after surgery. The stage of disease was reviewed by two independent clinicians using the 2010 American Joint Committee on Cancer (AJCC) TNM classification. Estimated GFR (eGFR) was calculated using the abbreviated Modification of Diet in Renal Disease (MDRD) equation recommended in the K/DOQI guideline [4] to replace the actual value of GFR with the equation “eGFR=175×(Scr)^-1.154^×(age)^-0.203^×(female×0.742), Scr (mg/dl), 1mg/dl=88.4 μmol/L, age (year old)”. According to the K/DOQI guidelines, chronic kidney dysfunction was defined as a GFR of<60 ml/min/1.73m^2^ for more than three months.

The postoperative incidence of CKD was calculated by Kaplan-Meier analysis and the life tables. The χ2 test, Student’s t test and rank-sum test were used to compare differences in qualitative and quantitative variables between patients with CKD (CKD group) and those without CKD (no CKD group). The independent risk factors for CKD occurrence were determined through logistic multivariate regression analysis. *P* values were 2-sided, with *P* < 0.05 defined as significant. Data analysis was conducted with IBM SPSS statistics 24.0 (SPSS, Inc., Chicago, IL).

## Results

**Fig. 1:**
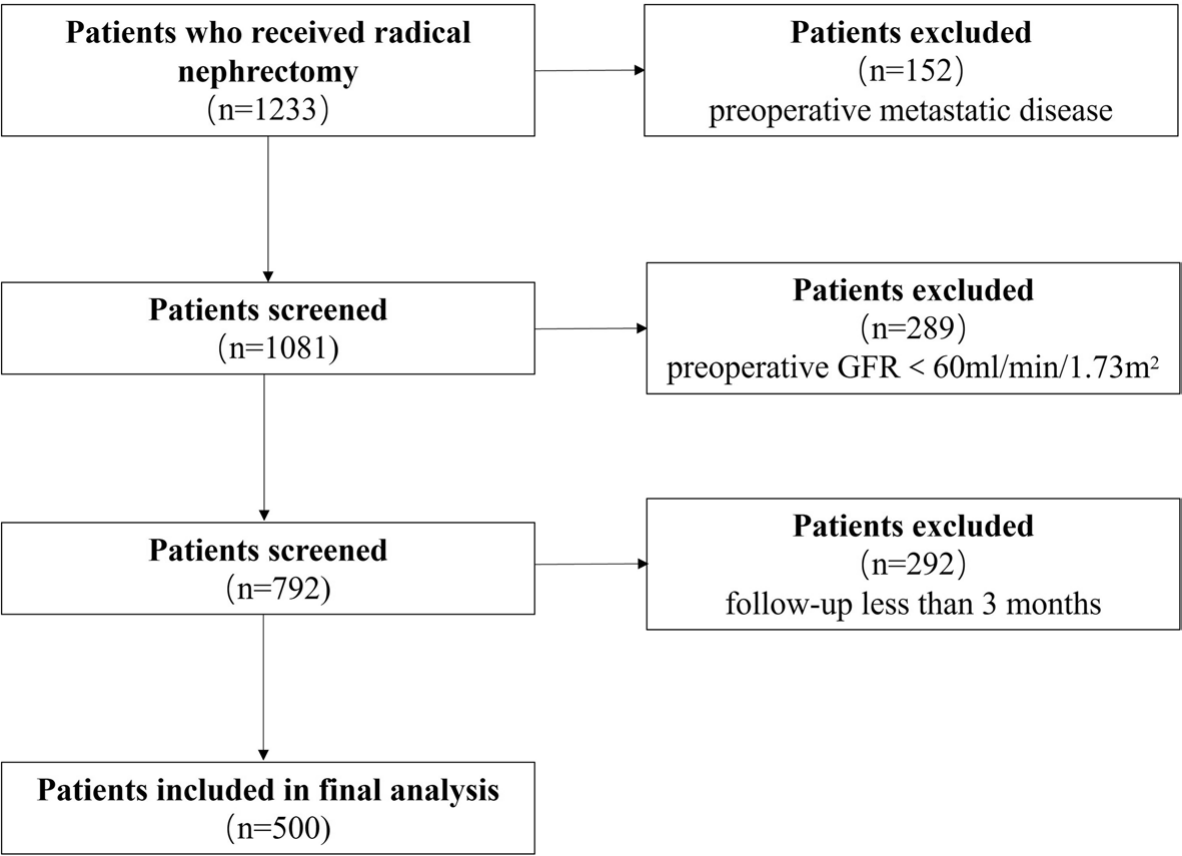
Flow diagram of the study selection process

A flow diagram of the study selection process is presented in Fig. 1. A total of 500 patients were enrolled in our study group, including 421 cases of clear cell carcinoma, 17 cases of papillary cell carcinoma, 20 cases of chromophobe cell carcinoma, 4 cases of acidophile cell adenoma and 38 cases of other pathology types. Patients were followed up for 3–96 months, with a median follow-up of 40 months. Patients lost to follow-up owing to poor compliance and prolonged assessment. Rate of lost to follow-up was 35.4% at 36 months, 59% at 60 months. A total of 189 patients developed postoperative CKD, with the 5-year cumulative incidence of CKD was 43.4%. Most CKD cases occurred within 1 year after surgery, with a median interval of 3 months. The cumulative incidence of CKD at 3 months, 1 year, 3 years and 5 years after surgery was 23.2, 36.0, 41.4 and 43.4% respectively.

We found that some patients in our cohort had improved renal function over time. A total of 130 patients were found to have CKD at the first follow-up visit (3 months after surgery), and the mean rate of decrease in eGFR was 27% compared to preoperative level. Gradual improvement in eGFR value observed in 40 CKD patients, and these patients had relatively high postoperative eGFR values. 37 of these 40 patients’ eGFR values were above 50 ml/min/1.73m^2^ when CKD was observed during the follow-up, and only 3 patients’ eGFR dropped to the level of 40-50 ml/min/1.73m^2^. 23 patients (13.3%) with normal immediate postoperative eGFR developed CKD within 5 years. Approximately 95 (50%) patients developed CKD if the contralateral normal kidney eGFR was less than 40 ml/min/1.73m^2^, and the probability increased to 70% if the contralateral normal kidney eGFR value was less than 30 ml/min/1.73 m^2^. Among all 500 patients, 155 patients were followed up for more than 6 years. The mean eGFR of 155 patients before surgery and 1 to 5 years after RN was 83.8, 69.4, 72.8, 72.1, 71.5, 73.1 ml/min/1.73 m^2^, and the mean rate of decrease in eGFR was 23.0, 19.9, 20.6, 21.6 and 20.2%. Among these 155 patients, if CKD did not occur within 5 years, no patients developed CKD afterwards.

**Fig. 2:**
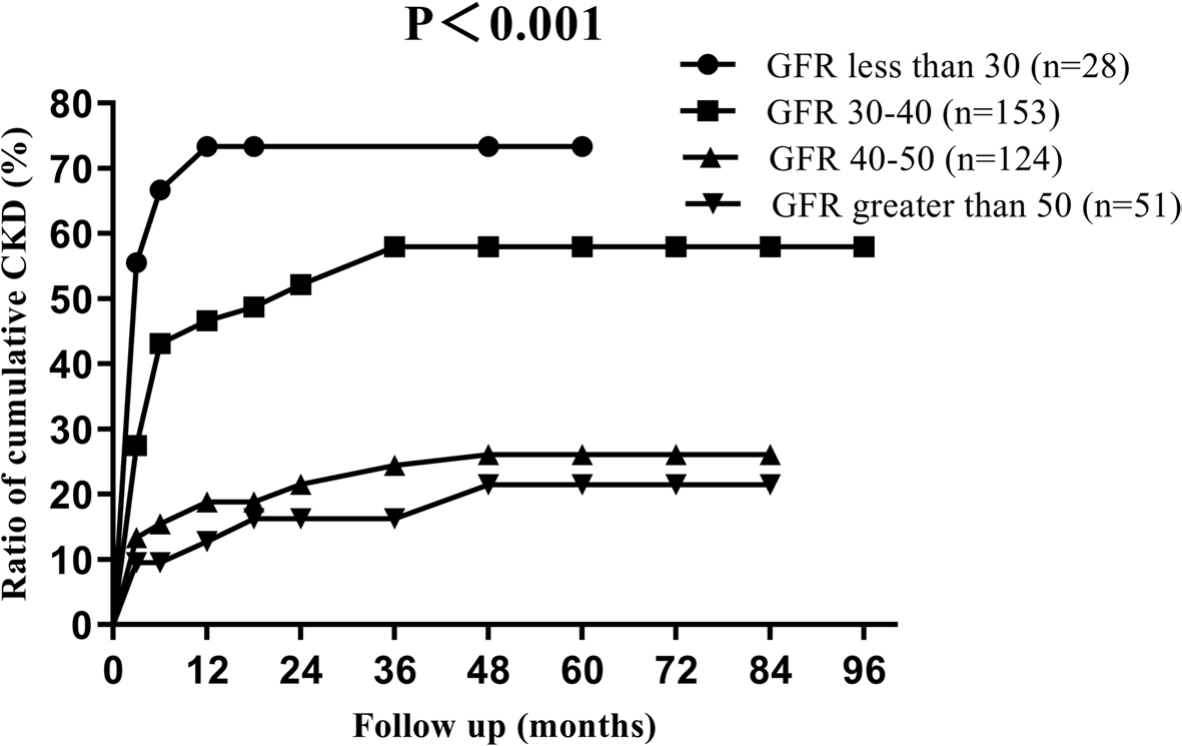
Ratio of cumulative incidence CKD in different preoperative contralateral GFR group. Contralateral GFR were measured by dynamic renal scintigraphy examination

**Fig. 3:**
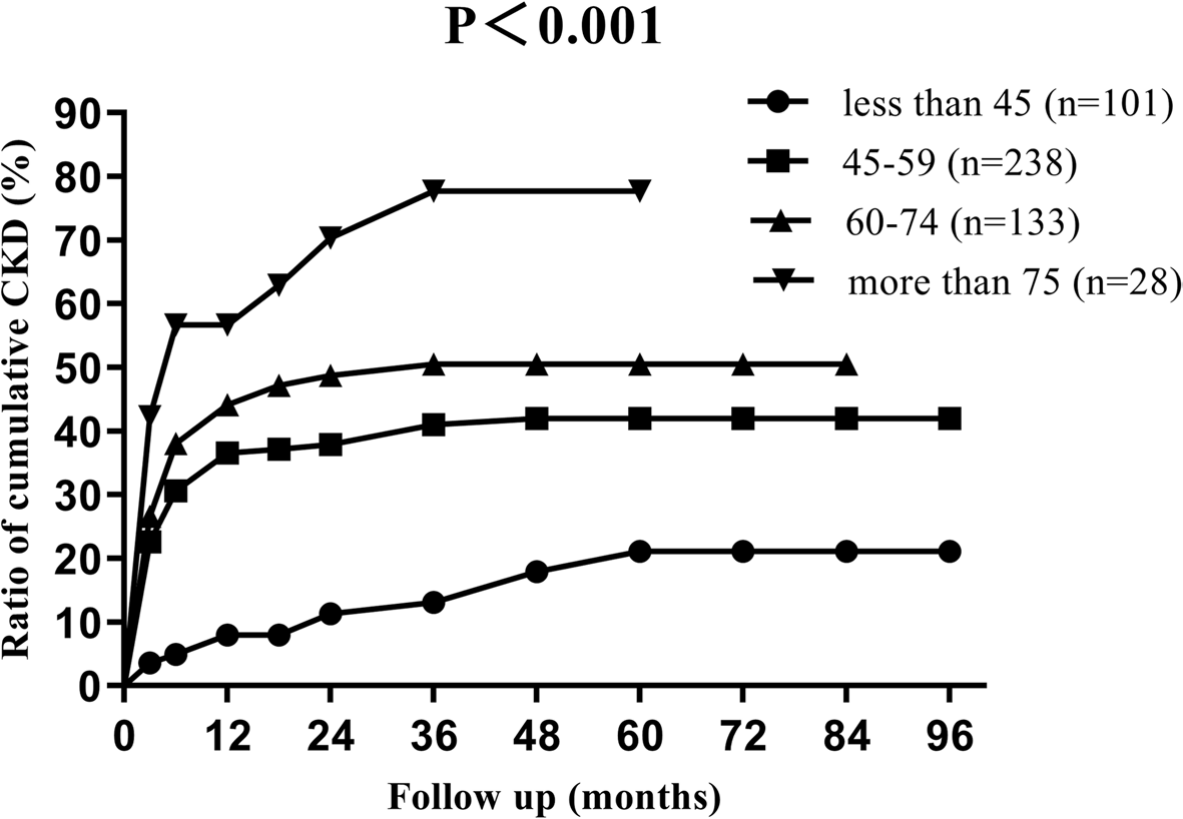
Ratio of cumulative incidence CKD in different age groups

**Fig. 4:**
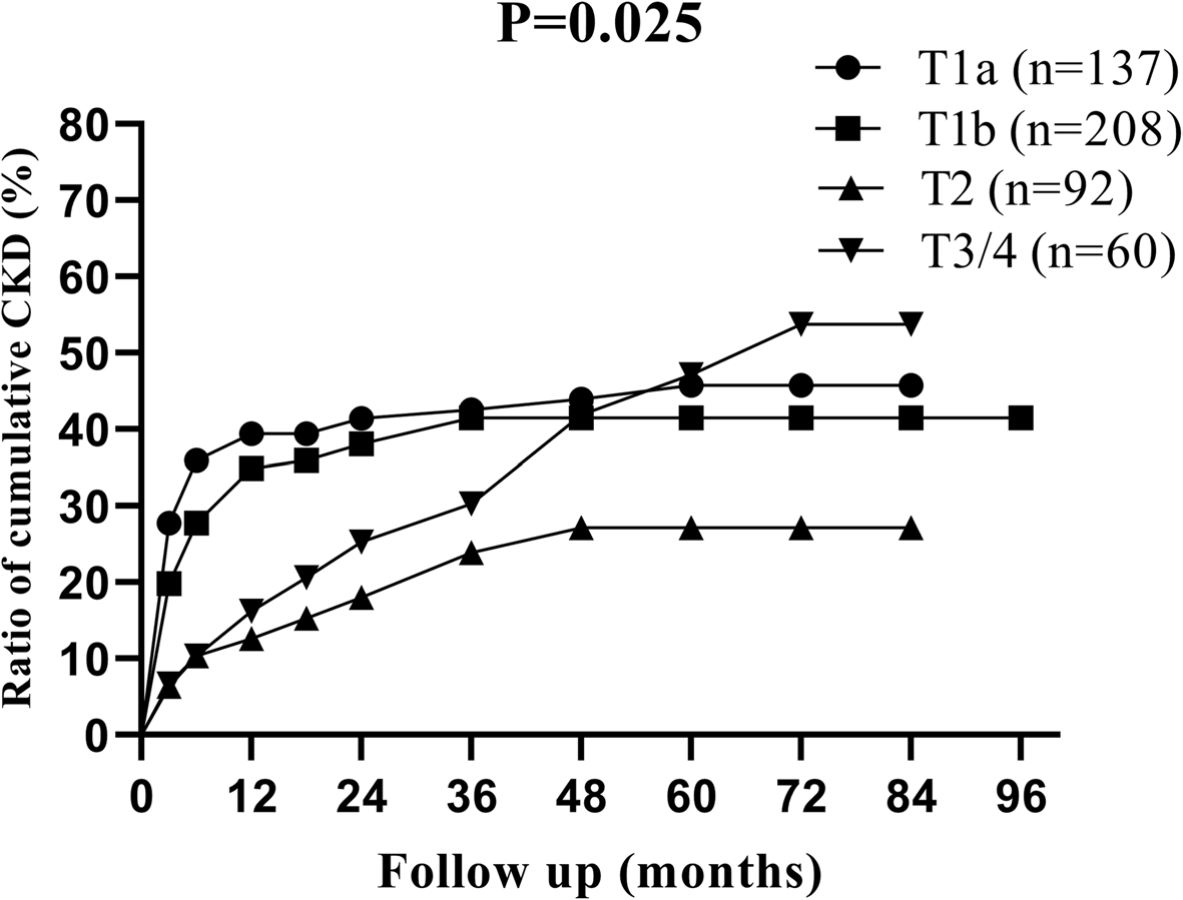
Ratio of cumulative incidence CKD in different T stages

We studied the cumulative incidences of CKD at different levels of preoperative contralateral GFR values, with the 5-years cumulative incidence of CKD as 72.0, 57.8, 27.5 and 24.5% respectively (Fig. 2). For different age levels, the 5-years cumulative incidence of CKD was 19.8, 42.1, 51.4 and 77.7%, respectively (Fig. 3). The incidence of CKD increased with increasing tumour size, with the 5-years cumulative incidence of CKD as 45.7, 41.5 and 27.2% for T1a, T1b and T2 stage, respectively (Fig. 4).

When comparing the difference between patients with CKD (CKD group) and patients without CKD (no CKD group), univariate analysis showed that these two groups were different in variables including age, sex, weight, preoperative GFR value, preoperative contralateral GFR value, β2-microglobulin, tumor size and Immediate postoperative eGFR value. (*P*<0.05, detailed in Table 1).

**Table 1:** Comparison of characteristics between CKD group and no-CKD group

characteristics	CKD group(*n* = 189)	no-CKD group(*n* = 311)	*P* value
	percentage or median (rang)		
age (year)	58(33–85)	51(27–85)	< 0.001
age group			< 0.001
≤ 44	17(9.0%)	84(27.0%)	
45–59	91(48.1%)	147(47.3%)	
60–74	63(33.3%)	70(22.5%)	
≥ 75	18(9.5%)	10(3.2%)	
sex			0.002
male	142(75.1%)	192(61.7%)	
female	47(24.9%)	119(38.3%)	
weight (Kg)	71(42–104)	68(41–101)	0.012
height (cm)	169(149–191)	167(143–190)	0.718
hypertension	64(33.9%)	80(25.7%)	0.052
diabetes	16(8.5%)	28(9.0%)	0.837
smoking history	52(27.5%)		0.470
proteinuria	29(15.3%)	55(17.7%)	0.497
kidney disease	51(27.0%)	90(28.9%)	0.638
anemia	10(5.3%)	20(6.4%)	0.603
total GFR value	75.5(40.3–173.2)	86.2(50.9–134.1)	< 0.001
contralateral GFR value	36.6(19.7–105.8)	42.4(25.5–66.2)	< 0.001
BMI (kg/m^2^)	24.8(16.0–36.8)	24.5(16.4–33.0)	0.080
fat	13(6.9%)	21(6.8%)	0.957
abnormal β2-microglobulin	36(19.0%)	40(12.9%)	0.031
hyperlipidemia	86(45.5%)	122(39.2%)	0.168
tumor size (cm)	4.7(2.0–13.0)	5.0(1.3–19.0)	0.028
T stage			0.223
T1	139(73.5%)	206(66.2%)	
T2	23(12.2%)	69(22.2%)	
T3	21(11.1%)	33(10.6%)	
T4	5(2.6%)	1(0.3%)	
unclassified	1(0.5%)	2(0.6%)	
Furhman grade			0.509
1	26(13.8%)	55(17.7%)	
2	117(61.9%)	144(46.3%)	
3	30(15.9%)	70(22.5%)	
4	6(3.2%)	11(3.5%)	
unclassified	10(5.3%)	31(10.0%)	
Immediate postoperative eGFR value^a^	51.6(25.6–92.4)	65.4(38.9–128.5)	< 0.001

^a^calculated within one week after surgery

Above significant factors were included in logistic multiple regression analysis and showed that age (OR = 1.038, 95%CI = 1.002–1.076), preoperative contralateral GFR value (OR = 0.934, 95%CI = 0.884–0.988) and Immediate postoperative eGFR value (OR = 0.892, 95%CI = 0.854–0.931) were independent risk factors. Patients had greater likelihood of CKD with increasing age，lower preoperative contralateral kidney GFR value or Immediate postoperative eGFR value.

## Discussion

RN is the classical treatment for localized renal cell carcinoma. Ideally, it should not cause CKD if the contralateral kidney is well preserved since the good renal function can be maintained by the remaining kidney in renal transplant patients. However, opponents proposed that kidney transplant patients were well selected and that their clinical outcomes could not be used to predict the outcome of RN patients, who are usually elderly and have decreased renal function preservation. Some of them have an underlying chronic disease, which will cause worsening kidney function over time [5]. In addition, pathology examination of radical nephrectomy always shows some kidney disease in the “no-tumor part” [6], which may be associated with CKD after RN [7]. Therefore, the probability of CKD after radical nephrectomy is often higher than expected.

Previously, Minato [8] found that the incidence of CKD 3-year after radical nephrectomy was 37%. Jeon [9] found that 41.7% T1a patients will suffer from CKD after nephrectomy. In our study, 43.4% of patients had CKD postoperatively after a relatively long follow-up. We also found that only 26 patients (13.7%) with normal immediate postoperative (within one week) eGFR develop CKD within 5 years. Xu [10] also showed that CKD incidence was much higher in patients who developed postoperative acute kidney injury than in patients who did not (18.64% vs. 5.94%), which means that acute kidney injury after nephrectomy may be a new nomogram to predict postoperative renal function.

Spontaneous recovery of kidney function was observed in 40 (21.2%) patients who had CKD after RN in our study. We consider that their kidney functions maybe haven’t been damaged seriously by functional compensation. Their postoperative eGFR values were usually more than 50 ml/min/1.73m^2^. In our study, we found that renal function worsened immediately after RN but improved thereafter, this finding is similar with Yokoyama’s study [8]. 155 patients were followed up for more than 6 years, among whom 78 patients did not develop CKD all the time. Thus, we found that if CKD did not occur within 5 years after surgery, it would not happen in following time. We considered that the kidney function would undergo compensatory recovery within 5 years after nephrectomy. Therefore, a routine examination for kidney function would not be necessary since the 5th year, possibly avoiding the unnecessary cost of the related items.

Previous studies showed that, age, race, sex, diabetes, hypertension, smoking, obesity, proteinuria and some clinical factors were risk factors for chronic kidney disease [11–14]. In our study, there were significant differences between the CKD group and the no-CKD group in age, sex, preoperative GFR value, preoperative contralateral GFR value, β2-microglobulin, tumor size and Immediate postoperative eGFR value. Multiple regression analysis showed that age, preoperative contralateral GFR value and Immediate postoperative eGFR value were independent risk factors of CKD postoperatively.

The preoperative evaluation of kidney function was very important for renal cancer patients. We consider preoperative dynamic renal scintigraphy was useful for evaluation of the contralateral kidney function and prediction the risk of acute renal failure after surgery. In our study, approximately 50% of patients developed postoperative CKD if their contralateral GFR value was less than 40 ml/min/1.73m^2^. If the contralateral GFR value was less than 30 ml/min/1.73m^2^, the incidence increased to nearly 70%.

According to the data from the United States Renal Data System [15], approximately 60% of patients with end stage renal disease (ESRD) were older than 75 years. In our study, both the univariate analysis and the multiple regression analysis showed that age was one of the risk factors of postoperative CKD (*P* < 0.001). Our stratified analysis showed that the risk of CKD increased with age. Moreover, there was a high incidence of CKD among patients older than 75 years old. So, the selection of proper surgery plan and preoperative suggestions seemed to be very important in older patients. Thus, we conclude that careful patient selection in elderly patient group was very important.

Our univariate analysis showed that “tumor size” was significantly different between CKD group and no-CKD group, however, multiple regression analysis not confirming that “tumor size” was an independent risk factor. In our study, 345 patients with T1 stage underwent radical nephrectomy, and 137 of them were T1a stage. Although studies have shown that the oncological outcome in terms of overall survival following partial nephrectomy equals that of radical nephrectomy in patients with T1 stage, clinically it is sometimes difficult to choose a surgical plan for renal tumors with relatively small volumes. It depends on the location of tumors (especially endophytic or parapelvic tumors), surgeon’s technique, and the patient’s requirement for surgical effect and safety. In our study, we analyzed the patients of T1-2N0M0 stage, with the 5-years incidence of CKD for the T1a, T1b, and T2 stages of 45.7, 41.5 and 27.2%, respectively, and the *P* value was 0.025. That is, the incidence of CKD in patients with localized renal cell carcinoma decreases with increased tumor size. In other words, the incidence of CKD was higher at the T1a stage than at the T1b stage. Several studies [16, 17] also reported a higher risk of postoperative CKD in patients with small tumors than those with larger tumors. As most renal cell carcinoma was slow-growing, we hypothesized that the compensation of the contralateral kidney was more developed before RN in larger ipsilateral tumor sizes, and patients were better able to tolerate the loss of nephrons during RN. Recently, Robert [18] found a significant interaction between age and tumor size, that is, tumor size may not have a protective effect on postoperative renal function, but this needs to be confirmed by further studies. Given that tumor size and age play important roles, partial nephrectomy may be a better choice for T1a stage or elder patients in order to decrease the incidence of postoperative CKD. However, it must be weighed against the increase in the perioperative risk especially for older individuals.

## Conclusions

Our study showed a significantly increased risk of CKD post RN, especially in older patients. The preoperative contralateral GFR value and Immediate postoperative eGFR value may be useful in evaluating the risk of developing postoperative CKD in renal cancer patients. These indicators can be used to assess the prognosis of renal function and develop appropriate follow-up plans for post radical nephrectomy patients. Therefore, preoperative renal function assessment and postoperative renal function follow-up are recommended.

## Data Availability

All data generated or analyzed during this study are included in this published article and available from the corresponding author.

## Abbreviations

eGFR: estimated glomerular filtration rate
CKD: Chronic kidney dysfunction
OR: Odds ratio
MDRD: Modification of Diet in Renal Disease
RN: Radical nephrectomy
AJCC: American Joint Committee on Cancer
Scr: Serum creatinine
K/DOQI: Kidney Disease Outcome Quality Initiative
